# Grandchild care, inadequate medical insurance protection, and inequalities in socioeconomic factors exacerbate childhood obesity in China

**DOI:** 10.3389/fpubh.2022.950870

**Published:** 2022-08-25

**Authors:** Jing Yang, Yun Shen, Yue Deng, Zangyi Liao

**Affiliations:** ^1^School of Public Administration, Hunan University, Changsha, China; ^2^School of Economics, Sichuan Agricultural University, Ya'an, China; ^3^Institute of Quality Development Strategy, Wuhan University, Wuhan, China; ^4^School of Political Science and Public Administration, China University of Political Science and Law, Beijing, China

**Keywords:** childhood obesity, grandchild care, medical insurance, socioeconomic factors, obesity inequalities, left-behind children

## Abstract

This study examines the influences of grandchild care and medical insurance on childhood obesity. Nationally representative longitudinal data—from the China Family Panel Studies 2010–2020—of 26,902 school-age children and adolescents aged 6–16 years and China's new reference standard (“WS/T586-2018”) are used to identify a child's obesity status. Using binary mixed-effects logistic regression models and the Blinder–Oaxaca decomposition method, this study explores the roots of obesity inequalities and finds that at least 15% of Chinese children aged 6–16 were obese in the 2010s. The logistic regression analysis results indicate that grandchild care, public medical insurance, and commercial medical insurance are key risk factors of child obesity. However, the influences are heterogeneous in different groups: Grandchild care and public medical insurance increase urban–rural obesity inequalities because of a distribution effect, and grandchild care may also exacerbate children obesity inequalities between left-behind and non-left-behind children owing to the event shock of parental absence. Inequalities in socioeconomic status (SES) factors such as income, education, and region also cause obesity inequalities. These results indicate that child obesity and its inequalities are rooted in multidimensional environmental inequalities, including medical protection policies and its benefit incidence; intergenerational behavior and family SES factors; and urban–rural and left-behind risk shocks. This study provides new evidence for the development of population-based interventions and equitable medical insurance policies to prevent the deterioration of child obesity among Chinese school-age children and adolescents.

## Introduction

As an important evaluation index of social development, children's nutrition and health have attracted much attention globally. At present, millions of children face nutritional imbalance, healthcare service shortages, and exposure to COVID-19. Some studies show that annually, malnutrition contributes to 3 million child deaths worldwide, and one in four children under age five have stunted growth due to nutritional problems (1). Meanwhile, obesity is a chronic nutritional disease caused by the excess of energy intake over energy consumption, and just like malnutrition, it is harmful to children's physical and mental health. Childhood obesity has become one of the most serious public health challenges in the 21st century (2), which is gradually transforming from a biological problem to a complex social problem (3, 4). Weight is impacted by genetic factors, environmental security, social status, and behavioral outcomes (5). Studies conducted in recent years have shown that during the COVID-19 lockdown, the rate of childhood obesity increased with the change in lifestyle (6, 7). However, compared with malnutrition, childhood obesity in developing countries is relatively overlooked (8). As poor countries escape from poverty traps and flee from famine, obesity rates rise (9). Although the overall rate of overweight and obesity among children in developing countries is lower than that in developed countries, obesity risk is accumulatively increasing at an alarming rate. In recent decades, China's situation is rapidly changing—from facing malnutrition to becoming the fastest growing obese population. Moreover, owing to population aging and parental migration (10), the phenomenon of intergenerational caring for grandchildren is relatively common (11). More importantly, the obesity costs of dependency burden and obesity-related medical care have increased astronomically, while the instability of family care continues to exacerbate the child obesity crisis (12). In 2020 and 2021, the COVID-19 pandemic severely affected the lives of children and young people worldwide, with public health measures taken to reduce community transmission of SARS-CoV-2, including unprecedented school closures and stay-at-home orders (13). As COVID-19 continues to spread globally, healthcare systems worldwide are overwhelmed, exacerbating the crisis of medical accessibility targeted at child obesity, thus making it urgent to address how to improve child obesity governance policies among generations.

### Children's health and its risk factors

A long-published study has documented that genetic factors (14) and the family socioeconomic environment (15–19) are risk factors affecting child health. Currie and Stabile (20) report that children with lower family socioeconomic status have difficulty recovering from adverse health shocks to a healthy state. Miller et al. (21) find the mediating effects of caregiver mental and physical health on children's mental health. Some studies document that mother-related characteristics of family functioning are also associated with children's mental health (22–24). Especially in early childhood, children's caregivers play an extremely important role in improving children's eating behavior and physical health (25). Aristides et al. (26) find that income inequities strongly contribute to healthcare inequalities and private health insurance, to inequalities of medicine use. Pickett et al. (27) find that social gradients and inequalities in almost all morbidities are the “causes of the causes” of child health. Few studies explore the health problems of left-behind children (28–31), finding that left-behind children are more likely to have poor health status due to parental absence. Some studies find that health services are beneficial to increasing the health-related outcomes of child health (32, 33). However, Paul et al. (34) find that maternal education remains an important determinant of child health outcomes in India, while a poor healthcare system weakens its effect. Although the impacts of medical factors on child health are widely discussed, few studies focus on the effects of multi-level medical systems and their benefit incidence heterogeneity on child obesity.

### Childhood obesity and family-based intervention

In recent years, the prevalence of childhood obesity has raised concerns because of the possible clinical and public health consequences (35). Childhood obesity leads to an increased risk of chronic diseases and seriously affects children's health (36). Previous studies find many factors affecting childhood obesity. Income and education socioeconomic status (SES) factors are inversely associated with child and adolescent obesity as measured by high body mass index (BMI) levels in developing countries (37–40). Zenab et al. (41) find that parental education, health insurance coverage, female gender, and language spoken at home other than Spanish were protective against overweight or obesity among children in the United States. Gopalan et al. (42) examine the spillover effects of parental public health insurance on the decrease of children's BMI, especially for girls. In some developing countries, especially in China, obesity risk is an important public health problem threatening child health (43, 44). Survey data show that between 1985 and 2010, the detection rates of obesity among male and female Chinese children and adolescents increased from 0.63 and 0.60% in 1985 to 11.60 and 5.59% in 2010, respectively (45). However, grandchild care exerts a certain negative impact on early childhood health of children aged 0–6 years (46). The spoiling effect of grandparents' indulgence on left-behind children produced negative effects on the children's BMI through an increase in unhealthy food consumption (47). Recent literature finds that childhood obesity also depends on emotional eating and family stability (48, 49). Moreover, recent studies of children have raised concerns about family parental factors and increased SES risk factors. Although the proportion of intergenerational-care families in China has been increasing in recent years (50), the influence of grandchild care on childhood obesity among school-age Chinese children and adolescents is underrepresented in the existing literature. Few studies focus on school-age children and adolescents and identify the link between intergenerational factors and childhood obesity (51). Thus, it remains unclear whether an interaction exists between grandchild care and child obesity in different groups. China continues to face special child obesity risks that are rooted in socioeconomic and population-based inequalities. Without an effective intervention, the prevalence of overweight and obesity among Chinese school-aged children and adolescents will reach 31.80% by 2030 (52). Since 2011, the Chinese government has enacted a series of “Child Development Plans” (2011–2020) and related health improvement policies to prevent childhood obesity effectively (53), and the trend of childhood obesity shows staged mitigation features. Although the prevention and treatment of obesity among children and adolescents were the focus in the 2010s (54, 55), the intervention policies targeting the roots of child obesity differences require further investigation.

### The present study

This study investigates the influences of grandchild care and medical insurance on child obesity using nationally representative longitudinal data between 2010 and 2020. We propose the following hypotheses: (1) Grandchild care increases the probability of childhood obesity; (2) medical insurance is positively associated with children's obesity risk; and (3) factors influencing child obesity are heterogeneous owing to group differences.

We take the following steps for our analysis: (1) investigate the recent prevalence trend of obesity rate among Chinese school-age children and adolescents and compare the differences among different groups; (2) examine the risk factors of childhood obesity inequalities and identify the associations among grandchild care, medical insurance, and child obesity; (3) decompose these factors' contributions and reveal how children are impacted by inequalities in different ways and the most important influencing factors, specifically, inequalities among urban–rural and left-behind children.

Our contributions to the literature are summarized as follows: 1) We explore the potential association between child obesity and grandchild care using new data from a nationally representative survey in China, the “China Family Panel Studies 2010~2020”; (2) we empirically identify the relationship between child obesity and medical insurance among Chinese school-age children and adolescents and evaluate the effectiveness of multi-level medical insurance programs; and (3) we decompose the factor inequalities of child obesity due to the urban–rural gap and left-behind experience. Our findings could help reduce the intergenerational spoil effect and enhance the health improvement effect of medical insurance on child obesity and then provide policy implications for improving population-based obesity intervention systems.

## Materials and methods

### Study population

The data for this study come from the CFPS (2010–2020) collected by the Institute of Social Science Survey, Peking University (https://opendata.pku.edu.cn/dataverse/CFPS). The CFPS is a ten-year longitudinal survey of a nationally representative cohort of Chinese communities, families, and individuals. In the 2010 baseline survey, the CFPS dataset included almost 15,000 families and 30,000 individuals within these families. Because of its systematically stratified sampling method and large-sample characteristics, these studies have provided one of the most nationally representative survey databases in China. The CFPS has the highest-quality survey database for children aged 0–16. A total of 26,902 school-age children and adolescents aged 6–16 years are included in our analysis.

### Statistical methods

We propose the following research hypotheses. First, grandchild care is associated with child obesity because of grandparental spoiling and unhealthy care practices. Second, medical insurance is related to the reduction of child obesity. Third, there is heterogeneity in the factors influencing child obesity because of environmental differences. In our investigation, we take the following steps: Step 1. We measure child obesity using the BMI index; Step 2. We analyze the influential factors of child obesity; Step 3. We apply the Blinder–Oaxaca decomposition method (BO) to compare the obesity inequality. Based on the regression results, we provide an in-depth discussion of the full empirical results.

A binary mixed-effects logistic regression model is employed to identify the influential factors of child obesity, in which the log odds of the binary outcome variable is modeled as a linear combination of the predictor variables when data are clustered or when both fixed and random effects exist^1^.


(1) $$\begin{array}{r} {\text{Obesity}}_{it}=IR_{it}\alpha +MS_{it}\beta +P_{it}\gamma +R_{it}\rho +\epsilon_{it} \end{array}$$

In Equation (1), the dependent variable *Obesity*_*it*_ is the obesity status of child *i* at time *t*. *IR*_*it*_represents grandchild care. *MS*_*it*_ represents medical insurance. *P*_*it*_ is a set of control variables that represent child-level (age and gender) and family-level (education and income) characteristics. *R*_*it*_ includes two-way fixed effects: region and year dummy variables. ε_*it*_ is the error term. A *p* < 0.05 means that the results are significant.

In addition, this study examines the heterogeneity of different factors influencing child obesity after distinguishing the types of Chinese school-age children and adolescents (e.g., urban vs. rural children and left-behind vs. non-left-behind children). Because traditional regression analysis is unable to derive directly the mean differences between groups, and the Shapley decomposition value method only yields the magnitude of the contribution of each factor in a linear regression model, neither of these models can decompose the differences between groups for models where the explanatory variable is a categorical variable. Therefore, we use BO for this analysis to investigate the coefficient effects of the urban–rural split and parental absence (or left-behind experience), and the characteristic effects of intergenerational parenting and health insurance on childhood obesity. BO is used to examine comparatively the determinants of childhood obesity differences among different groups. BO decomposes the differences in the dependent variables between groups into two components: changes in the distribution of levels of these determinants (*E*, explainable differences) and the consequence of the changes in the influence (*U*, unexplained differences).


(2) $$\begin{array}{r} E\left(Obesity_{A}\right)-E\left(Obesity_{B}\right)=X_{A}\beta_{A}-X_{B}\beta_{B}=\left(X_{A}-X_{B}\right)\beta^{*}\\ +X_{A}\left(\beta_{A}-\beta^{*}\right)+X_{B}\left(\beta_{B}+\beta^{*}\right) \end{array}$$

In Equation (2), *M* and *F* represent different groups. E(*Obesity*_*A*_) and E(*Obesity*_*B*_) represent the child obesity status in different groups, and *X*_*M*_ and *X*_*F*_ are the influential factors in the different obesity models. In our table, the term (X_A_-X_B_)β^*^ represents changes in the levels or values of a specific variable between different groups (distributional effect), while the term X_A_ (β_A_-β^*^)+X_B_ (β_B_-β^*^) captures the changes in the influence of a specific variable for determining obesity inequalities between these two groups (coefficient effect).

Following Powers et al. (56) and Yun (57), this study further extends Equation (2) to decompose and explain the estimated binary value of each coefficient.


(3) $$\begin{array}{r} E\left(Obesity_{A}\right)-E\left(Obesity_{B}\right)=E+U=\Sigma_{K=1}^{P}W_{\Delta X\beta_{k}}U\\ =\Sigma_{K=1}^{P}E_{k}+\Sigma_{K=1}^{P}U_{k} \end{array}$$

In Equation (3), *W*_Δ_x__*k*__represents the contribution share of the mean change of the *k* explanatory variable. *W*_Δ_x__β_k___reflects the contribution share of the coefficient estimated value change of the k explanatory variable.

### Measures

#### Measurement of childhood obesity

Child obesity is a dependent variable. We use BMI as the obesity indicator, which may dynamically reflect the childhood obesity status. BMI = weight (kg)/height^2^ (m^2^); it is a common clinical measurement indicator of physical health for assessing the status of child obesity. The recommended age-gender-specified BMI standard is used in our analysis to assess the trend of obesity rate for children and adolescents aged 6–16 years. The cutoff points of BMI are shown in Table 1. We use the determination criteria scale of the “WS/T586-2018 overweight and obesity screening of school-age children and adolescents,” established by the National Health Commission of China in 2018. Compared with the old criteria in versions 1985 and 2000, the recent standard, “WS/T 586-2018,” is more practical in terms of identifying the latest obesity prevalence in Chinese school-age children and adolescents.

**Table 1:** Cutoff points for obesity screening.

**Child's age**	**Boys**	**Girls**	**Child's age**	**Boys**	**Girls**
6	17.7	17.5	12.5	24.7	24.5
6.5	18.1	18.0	13	25.2	25.0
7	18.7	18.5	13.5	25.7	25.6
7.5	19.2	19.0	14	26.1	25.9
8	19.7	19.4	14.5	26.4	26.3
8.5	20.3	19.9	15	26.6	26.6
9	20.8	20.4	15.5	26.9	26.9
9.5	21.4	21.0	16	27.1	27.1
10	21.9	21.5	16.5	27.4	27.4
10.5	22.5	22.1	17	27.6	27.6
11	23.0	22.7	17.5	27.8	27.8
11.5	23.6	23.3	18	28.0	28.0
12	24.1	23.9			

#### Independent variables

**Grandchild Care**. Chinese families generally have a traditional culture of grandchild care, in which parents are busy with their work, and older people help families raise their grandchildren. Intergenerational care is an important way for Chinese families to care for their children. Consequently, the development and health of children may be affected by the intergenerational care from the grandparents. Based on the items in the CFPS questionnaire, specifically, “Who mainly takes care of the child during daytime or at night” or “Who takes care of the children during the month when the parents are not on vacation,” if the child's grandparents mainly take care of the grandchild, the value of *grandchild care* is set to 1 and 0 otherwise.

**Medical Insurance**. (1) For *public medical insurance*, insured = 1, and uninsured = 0. In China, the public insurance system (including resident basic medical insurance, new rural cooperative medical insurance, and other social medical items) is usually organized by the local and central governments. These provide institutional support for basic medical care for residents and cover medical costs incurred when individuals receive outpatient and inpatient care. (2) Regarding *commercial medical insurance*, the commercial medical insurance system is a supplementary medical insurance plan, which is managed by a commercial insurance company. Based on an item in the CFPS, specifically, “In the past 12 months, did the family buy any commercial medical insurance for the child?”, participated = 1, and did not participate = 0. (3) Regarding *medical utilization*, the variable is measured by the frequency of going to the hospital to see a doctor within a year.

**Covariates**. Child-level factors include age and sex. SES factors in our study include *income* and *education*. *Income* is measured by the household income status: if the family's income is greater than the median income, the value of the *income* variable is set to 1 and 0 otherwise. *Education* is represented by the value of the average education years of all the adult family members at the last interview. Region and year fixed effects are controlled for in every regression model. *Region* represents the survey address: east = 1, central = 2, and west = 3; *year* includes 2010, 2012, 2014, 2016, 2018, and 2020.

## Results

### Baseline characteristics and general trend

Table 2 shows that children cared for by their grandparents were more likely to suffer from obesity (21.96% are obese) compared with their counterparts. The chi-square (χ2) results of the chi-square test show significant differences in child obesity due to grandchild care (χ2 = 12.41, *p* < 0.001). The average obesity rate of children covered by public medical insurance was 15.68%, while that of uninsured children was 17.50%. The average obesity rate of children who participated in commercial medical insurance was also lower than those who did not participate. The χ2 test results document differences in child obesity due to public medical insurance (χ2 = 234.77, *p* < 0.001) and commercial medical insurance (χ2 = 31.89, *p* < 0.001).

**Table 2:** Baseline characteristics.

		**Child obesity rate, n(%)**		**P-value by using chi-square test**
		**No**	**Yes**	
Grandchild care	Yes	5,429 (78.04%)	1,528 (21.96%)	Pearson chi2(1)=12.41, *P* = 0.000
	No	17,130 (85.89%)	2,815 (14.11%)	
Basic Medical insurance	Insured	16,935 (84.32%)	3,150 (15.68%)	Pearson chi2(1) = 234.77, *P* = 0.000
	Uninsured	5,624 (82.50%)	1,193 (17.50%)	
Commercial medical insurance	Participated = 1	3,524 (86.86%)	533 (13.14%)	Pearson chi2(1) = 31.89, *P* = 0.000
	Unparticipated = 0	19,035 (83.32%)	3,810 (16.68%)	

Table 3 presents the descriptive statistics, including the means, standard deviations (SDs), and range of all the variables included in the analysis. The average grandchild care for the children surveyed was more than one in four. The participation rate in basic health insurance was more than 70%, while the participation rate in commercial health insurance was only 15%. The results of the chi-square test or analysis of variance (ANOVA) show significant obesity differences based on the demographic variables (*p* < 0.05).

**Table 3:** Demographic variables.

	**2010 (*n =* 4,967) Mean (SD) or n (%)**	**2012 (*n =* 4,460) Mean (SD) or n (%)**	**2014 (*n =* 4,514) Mean (SD) or n (%)**	**2016 (*n =* 4,484) Mean (SD) or n (%)**	**2018 (*n =* 4,825) Mean (SD) or n (%)**	**2020 (*n =* 3,652) Mean (SD) or n (%)**	**2010~2020 (*n =* 26,902) Mean (SD) or n (%)**
**Grandchild care**	
Grandparent-raised = 1	1,139 (22.93%)	1,075 (24.10%)	1,185 (26.25%)	1,261 (28.12%)	1,510 (31.30%)	787 (21.55%)	6,957 (25.86%)
Non-grandparent-raised = 0	3,828 (77.07%)	3,385 (75.90%)	3,329 (73.75%)	3,223 (71.88%)	3,315 (68.70%)	2,865 (78.45%)	19,945 (74.14%)
**Public medical insurance**	
Insured = 1	3,145 (63.32%)	2,435 (54.60%)	3,088 (68.41%)	4,021 (89.67%)	4,368 (90.53%)	3,028 (82.91%)	20,085 (74.66%)
Uninsured = 0	1,822 (36.68%)	2,025 (45.40%)	1,426 (31.59%)	463 (10.33%)	457 (9.47%)	624 (17.09%)	6,817 (25.34%)
**Commercial medical insurance**	
Participated = 1	875 (17.62%)	567 (12.71%)	676 (14.98%)	539 (12.02%)	637 (13.20%)	763 (20.89%)	4,057 (15.08%)
Unparticipated = 0	4,092 (82.38%)	3,893 (87.29%)	3,838 (85.02%)	3,945 (87.98%)	4,188 (86.80%)	2,889 (79.11%)	22,845 (84.92%)
Medical utilization (range 0~60)	1.39 (2.37)	0.93 (1.94)	1.67 (3.64)	1.71 (3.10)	1.74 (3.69)	1.17 (2.39)	1.45 (2.96)
Age (range 6~16)	10.59 (2.92)	10.41 (2.92)	10.37 (2.90)	10.13 (2.86)	10.25 (2.88)	10.31 (2.85)	10.35 (2.89)
**Gender**	
Male	2,571 (51.76%)	2,335 (52.35%)	2,384 (52.81%)	2,430 (54.19%)	2,583 (53.53%)	1,950 (53.40%)	14,253 (52.98%)
Female	2,396 (48.24%)	2,125 (47.65%)	2,130 (47.19%)	2,054 (45.81%)	2,242 (46.47%)	1,702 (46.60%)	12,649 (47.02%)
**Income**	
High-income	2,571 (51.76%)	2,335 (52.35%)	2,130 (52.81%)	2,430 (54.19%)	2,583 (53.53%)	1,950 (53.40%)	13,490 (50.14%)
Low-income	2,396 (48.24%)	2,125 (47.65%)	2,384 (47.19%)	2,054 (45.81%)	2,242 (46.47%)	1,702 (46.60%)	13,412 (49.86%)
Education (range 0~30)	5.98 (3.96)	6.18 (3.68)	5.86 (3.45)	6.17 (3.47)	6.87 (3.21)	7.27 (3.41)	6.36 (3.58)
**Region**	
Eastern	1,766 (35.55%)	1,594 (35.74%)	1,502 (33.27%)	1,448 (32.29%)	1,652 (34.24%)	1,301 (35.62%)	9,263 (34.43%)
Middle	1,526 (30.72%)	1,390 (31.17%)	1,477 (32.72%)	1,462 (32.60%)	1,545 (32.02%)	1,143 (31.30%)	8,543 (31.76%)
Western	1,675 (33.72%)	1,476 (33.09%)	1,535 (34.01%)	1,574 (35.10%)	1,628 (33.74%)	1,208 (33.08%)	9,096 (33.81%)

Chi-square test for heterogeneity(categorical variables), ANOVA test for differences in the mean (continuous normally distributed variables). SD, standard deviation.

**Figure 1:**
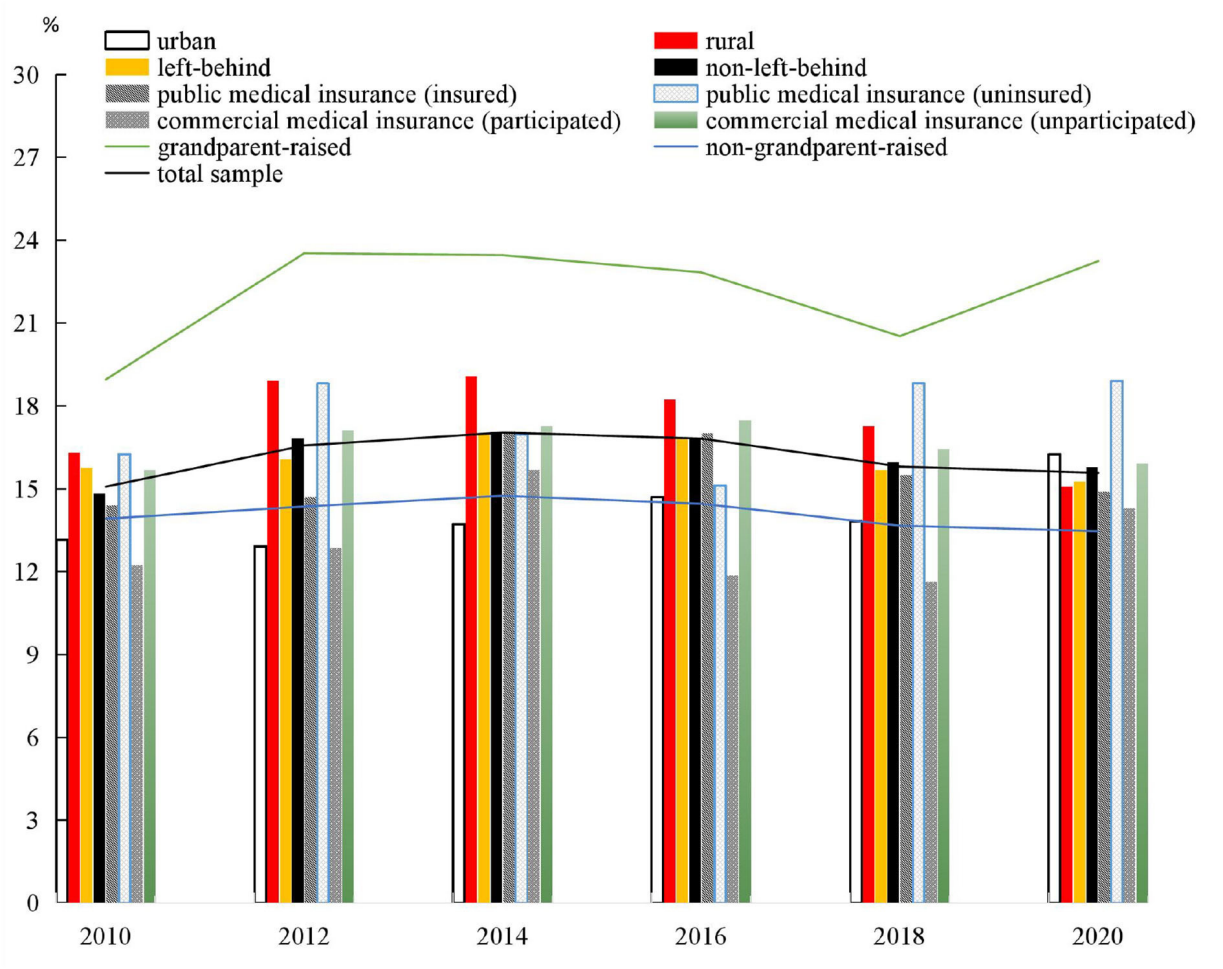
Child obesity rate during 2010–2020(%).

Figure 1 shows the prevalence change in child obesity between 2010 and 2020. We find that at least 15% of children aged 6–16 years were obese in the past 10 years. The obesity rate of grandparent-raised children rose from 18.96 to 23.25% from 2010 to 2020, while the non-grandparent-raised children's obesity rate was always below 15%. The results of the trend analysis indicate that the obesity rate of children raised by their grandparents is greater than that of the non-grandparent-raised group. Between 2010 and 2020, the obesity rate of children that did not participate in the commercial medical insurance was greater than that of their counterparts. Meanwhile, since 2016, the obesity rate of the group that participated in public medical insurance was greater than that of their counterparts. In addition, before 2020, the obesity rate of urban children was lower than that of children from rural areas. However, the obesity rates of left-behind and non-left-behind groups stayed largely the same since 2010. Thus, the results show inter-group differences in child obesity.

### Grandchild care

Table 4 shows that the impact of grandchild care on child obesity is significant (OR = 1.15, 95% CI: [1.07, 1.25], p = 0.000). Compared with the case of non-grandparent-raised children, grandchild care is significantly positively associated with child obesity. A possible explanation is that these grandparents had difficulty providing reasonable care for their grandchildren because of the decline in their bodily functions. In addition, majority of these grandparents likely had poor health awareness (58), such that they tended to provide excessive food to the child. Unhealthy grandchild-caring habits such as unhealthy diets, too much television, too little activity, and too little sleep were likely detrimental to the Chinese children's weight management.

**Table 4:** Influencing factors of child obesity.

	**Odds Ratio**	**Std. Err**.	**z**	**P>|z|**	**[95% confidence interval]**	
Grandchild care	1.1547	0.0448	3.71	0.000	1.0702	1.2460
Public medical insurance	0.8735	0.0377	−3.13	0.002	0.8026	0.9506
Commercial medical insurance	0.8397	0.0453	−3.24	0.001	0.7555	0.9333
Medical utilization	0.9655	0.0063	−5.36	0.000	0.9532	0.9780
Age	0.9159	0.0531	−1.51	0.130	0.8175	1.0262
Age-square	0.9866	0.0030	−4.51	0.000	0.9808	0.9924
Gender	1.3227	0.0479	7.72	0.000	1.2320	1.4200
Income	0.8797	0.0338	−3.33	0.001	0.8158	0.9486
Education	0.9439	0.0054	−10.16	0.000	0.9334	0.9545
Middle	1.0290	0.0476	0.62	0.536	0.9399	1.1267
Western	1.3710	0.0627	6.90	0.000	1.2534	1.4997
Year FE	YES	YES	YES	YES	YES	YES
Constant	1.9573	0.5401	2.43	0.015	1.1397	3.3614

### Medical insurance

Over 74% of children were covered by public medical insurance, while over 15% of the children participated in commercial medical insurance. Uninsured children were more likely to be obese than insured children (OR = 0.87, 95% CI: [0.80, 0.95], p = 0.002; OR = 0.84, 95% CI: [0.76, 0.93], p = 0.001). The results indicate that although most Chinese children had commercial medical insurance, the implementation of a multi-level medical insurance system could decrease the probability of Chinese school-age children and adolescents becoming obese. It is widely discussed that China's various types of medical insurance are useful in alleviating the cost of obesity-related medical care, fostering the healthy eating behaviors, and increasing the level of children's medical use, which may eventually reduce child obesity. Since the medical insurance reform was implemented in the 2010s, the increasing supportive policies for healthcare providers have improved the medical environment for insured children. Medical utilization is also a risk factor affecting child obesity (OR = 0.97, 95% [CI: 0.95, 0.98]), which indicates that a lesser frequency of medical utilization is associated with a higher prevalence of obesity, suggesting that increasing the accessibility and quality of children's medical utilization may help in timely mitigating obesity risks and reducing the damage of chronic diseases. More importantly, the medical advice provided by doctors also widens both the child's and parents' awareness of obesity risks, leading to an improvement in the child's daily weight management. Thus, it is necessary to improve the social insurance system and develop a specific commercial medical insurance for child obesity management.

### Covariates

The study finds that the child's gender is associated with elevated levels of BMI. In China, boys are usually spoiled by the family, which may result in gender differences in childhood obesity (OR = 1.32, 95% CI: [1.23, 1.42], *p* < 0.001). Child obesity is influenced significantly by child age, but the impact of age-square shows an inverted U curve (OR = 0.99, 95% CI: [0.98, 0.99], *p* < 0.001). The results also show an obvious relationship between endowment disadvantage and child obesity. SES factors (income and education) are significantly associated with an elevated obesity status (OR = 0.88, 95% CI: [0.82, 0.95], p = 0.001; OR = 0.94, 95% CI: [0.93, 0.95], *p* < 0.001, respectively), which indicates that a lower SES is associated with a higher obesity rate. One explanation may be that children with a lower family SES tend to choose foods with higher calorie, which in turn leads to child obesity. Another possible explanation is that families with lower SES invest less resources in weight management for the child. In addition, child obesity is higher in the western economically underdeveloped region than in the eastern developed provinces (OR = 1.37, 95% CI: [1.25, 1.50], *p* < 0.001).

### Obesity differences and blinder–oaxaca decomposition

#### Heterogeneity analysis of urban–rural differences

The results of the study show urban–rural differences in the influential factors of child obesity (Table 5). Risk factors that influence obesity among urban children include grandchild care (OR = 1.21, *p* < 0.01), public medical insurance (OR = 0.85, ***p*<** 0.05), medical utilization (OR = 0.97, *p* < 0.01), age-square (OR = 1.00, p = 0.001), gender (OR = 1.53, *p* < 0.001), education (OR = 0.96, *p* < 0.001), and a western location (OR = 1.29, p = 0.001). Risk factors influencing rural children's obesity include grandchild care (OR = 1.13, *p* < 0.05), commercial medical insurance (OR = 0.97, *p* < 0.001), medical utilization (OR = 1.21, *p* < 0.001), age-square (OR = 0.98, *p* < 0.001), income (OR = 0.86, *p* < 0.001), education (OR = 0.93, *p* < 0.01), and a western location (OR =1.38, *p* < 0.001). The results show that public medical insurance has a more significant effect on urban than on rural children, but we have not confirmed the statistically significant effect of commercial medical insurance on reducing urban children's obesity risk. Thus, the association between medical insurance and child obesity is heterogeneous for urban and rural children. The disparity in healthcare conditions may contribute to the differences in child obesity status and its influential factors when comparing rural and urban children. Medical utilization is significantly associated with both urban and rural children, while the income and age-square variables only have significant effects on rural children. This means that intervention policies should focus either on the improvement of SES or the medical environment. Our study confirms that the effects of public medical insurance are significant only on urban children's obesity, while the effects of commercial medical insurance and income are significant only on rural children. Thus, for urban children, the aim should be to improve public medical insurance and medical utilization, while for the latter, it should be to increase the commercial medical insurance coverage and income status. Therefore, policies are urgently needed to combat these obesity inequalities, and population-based and family-focused interventions on childhood obesity should be implemented in a coordinated manner.

**Table 5:** Obesity difference between urban and rural children.

	**Urban children (*N =* 10,869)**				**Rural children (*N =* 16,033)**			
	**Odds Ratio**	***P* > |z|**	**[95% confidence interval]**		**Odds Ratio**	***P* > |z|**	**[95% confidence interval]**	
Grandchild care	1.2065	0.003	1.0654	1.3663	1.1285	0.014	1.0246	1.2429
Public medical insurance	0.8469	0.017	0.7392	0.9703	0.9001	0.058	0.8074	1.0034
Commercial medical insurance	0.9404	0.413	0.8117	1.0894	0.7365	0.000	0.6323	0.8579
Medical utilization	0.9696	0.005	0.9489	0.9907	0.9654	0.000	0.9501	0.9809
Age	0.7419	0.001	0.6228	0.8837	1.0661	0.406	0.9167	1.2397
Age-square	1.0003	0.956	0.9914	1.0092	0.9769	0.000	0.9693	0.9847
Gender	1.5318	0.000	1.3648	1.7193	1.2045	0.000	1.1006	1.3183
Income	0.9198	0.189	0.8119	1.0419	0.8626	0.003	0.7828	0.9507
Education	0.9567	0.000	0.9410	0.9726	0.9345	0.000	0.9195	0.9498
Middle	1.0691	0.322	0.9368	1.2202	0.9913	0.890	0.8758	1.1220
Western	1.2899	0.001	1.1147	1.4927	1.3789	0.000	1.2266	1.5501
Year FE	Yes				Yes			
Constant	3.3374	0.005	1.4305	7.7862	1.2542	0.532	0.6159	2.5539

#### Left-behind event shock

Compared with non-left-behind children, the phenomenon of intergenerational care in China among left-behind children is more widespread. Specifically, the data show that in 2020, more than 67% of left-behind children were mainly cared for by their grandparents. In our study, left-behind children are those whose parents stayed outside the home for a long time and rarely went back home to care for their children. A child is considered left-behind in this study if one or both parents did not live with them for <6 months in 1 year or left home for at least 1 month. Because of the shock of being left behind, the instability of the family structure may exacerbate the childcare crisis, leading to parents spending less time caring for left-behind children, and the raising burden being transferred to the elderly. Because of parental absence and grandchild care vulnerability, the influence of grandchild care on child obesity may differ between left-behind and non-left-behind children.

The results in Table 6 show that the factors influencing left-behind children's obesity include grandchild care (OR = 1.27, *p* < 0.001), medical utilization (OR = 0.96, *p* < 0.001), age-square (OR = 0.99, *p* < 0.01), gender (OR = 1.18, *p* < 0.01), income (OR = 0.81, p = 0.001), education (OR = 0.95, *p* < 0.001), and a western location (OR = 1.43, *p* < 0.001). The factors influencing non-left-behind children's obesity include public medical insurance (OR = 0.85, *p* < 0.05), commercial medical insurance (OR = 0.85, *p* < 0.01), medical utilization (OR = 0.97, *p* < 0.05), age-square (OR = 0.99, p = 0.001), gender (OR = 1.41, *p* < 0.001), education (OR = 0.94, *p* < 0.001), and a western location (OR = 1.34, *p* < 0.001).

**Table 6:** Child obesity difference between left-behind children and non-left-behind children.

	**Left-behind children (*N =* 9,916)**				**Non-left-behind children (*N =* 16,986)**			
	**Odds Ratio**	**P > |z|**	**[95% confidence interval]**		**Odds Ratio**	**P > |z|**	**[95% confidence interval]**	
Grandchild care	1.2673	0.000	1.1221	1.4314	1.0994	0.085	0.9871	1.2244
Public medical insurance	0.9172	0.245	0.7929	1.0610	0.8490	0.002	0.7646	0.9427
Commercial medical	0.8232	0.051	0.6773	1.0006	0.8480	0.011	0.7472	0.9624
insurance
Medical utilization	0.9607	0.000	0.9419	0.9798	0.9690	0.000	0.9526	0.9856
Age	0.9085	0.321	0.7516	1.0981	0.9092	0.193	0.7878	1.0493
Age-square	0.9862	0.005	0.9767	0.9958	0.9874	0.001	0.9801	0.9947
Gender	1.1759	0.007	1.0444	1.3239	1.4150	0.000	1.2939	1.5474
Income	0.8110	0.001	0.7156	0.9191	0.9297	0.136	0.8448	1.0232
Education	0.9490	0.000	0.9304	0.9680	0.9388	0.000	0.9260	0.9518
Middle	0.9848	0.847	0.8422	1.1515	1.0656	0.269	0.9522	1.1925
Western	1.4326	0.000	1.2255	1.6748	1.3447	0.000	1.2045	1.5013
Year FE	YES				YES			
Constant	1.8516	0.183	0.7484	4.5807	2.0295	0.041	1.0290	4.0027

We confirm that grandchild care increases the obesity risk of left-behind children, but there is no significant association between grandchild care and child obesity for non-left-behind children. Moreover, public and commercial medical insurance may lower the child obesity rate only for non-left-behind children (OR <1, *p* < 0.05), while the effects on left-behind children are not significant. This indicates no significant protective effects of medical insurance in reducing the risk of obesity among left-behind children in China. Income is only associated with left-behind children's obesity, while the effect of income on non-left-behind children is not significant. Thus, the obesity effects of grandchild care, medical insurance, and other SES factors are heterogeneous.

#### Decomposition of childhood obesity inequalities

We further apply BO to identify the contribution share of the above-mentioned factors on the differences in child obesity. The explainable endowment effect reflects the percentage increase in the probability of urban children being obese when they have similar endowment characteristics as rural children. The results show that the obesity rate of rural children is higher than that of urban children. The decomposition of the obesity gap shows that the unexplained share related to differences in individual characteristics is −0.0141, or 39.90% of the total wage gap. The decomposition of the obesity gap shows that the explained share associated with differences in individual characteristics is −0.0212, or 60.10% of the total wage gap. The urban–rural decomposition results in Table 7, panel A, indicate that the proportion of endowment differences is larger than the coefficient differences (unexplained component). The obesity gap between urban and rural children is largely due to the explained part of the BO.

**Table 7:** Decomposition of child obesity differences due to characteristic endowment.

	**Panel A: Reference group (Urban children)**		**Panel B: Reference group (Left-behind children)**	
	**vs. Comparison group (rural children)**		**vs. Comparison group (non-left-behind children)**	
	**C**	**Pct. (%)**	**C**	**Pct. (%)**
Overall difference	−0.0353(0.000)	100	0.0005(0.902)	100
Explainable differences	−0.0212(0.000)	60.10	0.0089(0.000)	1644.70
Unexplained differences	−0.0141(0.010)	39.90	−0.0083(0.095)	−1544.70
**Due to Difference in Characteristics**
Grandchild care	−0.0005(0.003)	1.42	0.0088(0.000)	1633.16
Public medical insurance	−0.0001(0.025)	0.32	−0.0003(0.250)	−63.79
Commercial medical insurance	−0.0006(0.455)	1.69	0.0015(0.062)	269.43
Medical utilization	0.0002(0.003)	−0.61	−0.0015(0.000)	−280.39
Age	0.0005(0.000)	−1.51	−0.0113(0.002)	−2093.15
Age-square	−0.0002(0.149)	0.69	−0.0047(0.322)	−871.74
Gender	−0.0001(0.000)	0.32	−0.0001(0.005)	−21.15
Income	−0.0030(0.139)	8.40	0.0027(0.002)	497.75
Education	−0.0133(0.000)	37.72	0.0095(0.000)	1753.93
Middle area	0.0002(0.407)	−0.56	−0.0002(0.792)	−43.99
Western area	−0.0050(0.004)	14.16	0.0019(0.000)	351.23

The results related to the coefficient effect (unexplainable part) are not listed.

Considering the decomposition of the different variables in Table 7, the significant contribution shares of some of the endowment effect on child obesity are significant (*p* < 0.05), including grandchild care (C = −0.0005, *p* = 0.003), public medical insurance (C = −0.0001, *p* = 0.025), gender (C = −0.0001, *p* =0.000), education (C = −0.0133, *p* = 0.000), and a western location (C = −0.005, *p* = 0.004). The factors above are both negative components that can increase the obesity difference between urban and rural children. Overall, the obesity rate is lower for urban than for rural children, while public medical insurance is higher for urban than for rural children. Thus, identifying an uninsured child is important to alleviate the obesity difference among urban and rural Chinese children. In addition, grandchild care, which is negatively associated with child obesity, contributes to widening the inequalities in obesity between urban and rural children, as urban children have lower levels of intergenerational parenting than rural children. The endowment differences in education and gender are also major contributors to the changes in obesity differences between urban and rural children. Moreover, medical utilization (C = 0.0002, *p* = 0.003) and child age (C = 0.0005, *p* = 0.000) importantly contribute to the decrease in the obesity inequalities between urban and rural children. In China, the difference in medical utilization may decrease the obesity difference resulting from the integration of China's urban–rural public welfare system. The urban–rural differences in obesity among Chinese school-age children and adolescents shrink with age because of physiological factors.

In Table 7, panel B, the contribution share of the endowment effect is slightly larger than that of the coefficient effect between left-behind and non-left-behind children. Specifically, medical utilization (C = −0.0015, *p* = 0.000), age (C = −0.0113, *p* = 0.002), and gender (C = −0.0001, *p* = 0.005) are significantly negative, owing to the endowment difference. The results indicate that these are the main factors for reducing the obesity difference between non-left-behind and left-behind children. However, the influence of grandchild care (C = 0.00881, *p* = 0.000), income (C = 0.0027, *p* = 0.002), education (C = 0.0095, *p* = 0.000), and a western location (C = 0.0019, *p* = 0.000) is significantly positive, indicating that the differences in these variables exacerbate the obesity inequality. The results show that changes in the distribution of grandchild care in different families are the major contributors to changes in children obesity inequalities because of the distribution effect. Because the incidence of obesity and grandchild care in left-behind children is higher than that in non-left-behind children, unbalanced grandchild care behavior may worsen the obesity inequality in left-behind children. Moreover, although the alleviation effect of education on child obesity for left-behind children and non-left-behind children is both significant, the inequality in education is still a source of obesity difference. The previous results suggest that income has a suppressive effect on obesity only for left-behind children, not for non-left-behind children; therefore, the income disparity may exacerbate obesity inequalities between left-behind and non-left-behind children. Moreover, medical utilization can reduce the obesity difference. In addition, the effects reflected by individual variables (age and gender) can decrease the inequalities in child obesity.

## Discussion

Over recent decades, the overweight and obesity situation among Chinese children has become serious. According to the “WHO child growth standards (2006)” for school-aged children and adolescents, the results of the “China childhood obesity report (2017)” meant that the obesity rate among Chinese children aged 7–18 years in 1985 and 2000 was 0.5 and 4.6%, respectively, and the predictive value of obesity rate may reach only 8.5% in 2020 (10). However, using the “WS/T586-2018” as a reference, Song et al. (59) find that the obesity rate rose from 2.3 to 15.2% from 1991 to 2015, and the obesity rate for girls rose from 2.5 to 10.1%, which is worse than what Ma et al. (10) predicted. Our findings are closer to those of Song et al. (59). We document that at least 15% of Chinese children aged 6–16 years were obese during 2010–2020. Although the difference in the results may be related to the measurement tools and data sources, the obesity risk is clearly becoming a serious public problem threatening child development; thus, it is necessary to take urgent obesity control measures from different perspectives.

Our results indicate that left-behind children and rural children who are cared for by grandparents have a higher probability of being obesity. Some studies find a consistent conclusion that parental absence made left-behind children in rural areas become a high-risk group prone to nutrition and health problems (60, 61). A study confirms that the mechanisms by which grandchild care affects the physical health of left-behind children are complex (47). Although grandchild care may have some positive effects in offsetting children's psychological loss due to parental absence (50), the negative effect of grandchild care on child obesity remains dominant in our study. We further determine that differences exist in the intergenerational mechanisms on child obesity among different groups, and we believe that policies that support and optimize the behaviors of intergenerational care may be a key measure for preventing children's obese status.

In fact, child obesity is not caused only by child-related factors. Aside from child-level differences, child obesity is also closely related to socio-political factors (18). Some studies have confirmed that social medical insurance in rural areas could reduce preventive savings by increasing the uncertainty of future medical expenditures, increase consumption, and improve nutrition intake and the child's health (62, 63). Our study also finds that having medical insurance can reduce the risk of child obesity, especially for urban and non-left-behind children. However, we find no significant association between public medical insurance and child obesity for rural and left-behind children, while public medical insurance is an important factor to the obesity inequalities between urban and rural children. The outcome disparities reflect that the inequalities in the benefits of medical insurance are still obvious because of the differences in the children's growth environment, which means that the role of public medical insurance in protecting the physical health of vulnerable children (rural and left-behind children) is inefficient. We find that commercial medical insurance is significantly associated with the obesity status of rural children. Therefore, the family and political environments are important to the prevention of child obesity. This means that governments at all levels should further improve the design of the medical insurance systems for different children and strengthen the complementary role of commercial health insurance to prevent the obesity risk of rural children. In addition, policies supporting primary grandchild caregivers should be made to prevent child obesity.

In line with previous research (64–66), we find that the prevalence of child obesity is higher in families with a lower SES. Especially for rural and left-behind children, family income and education are important in reducing childhood obesity rates. A study finds an inverse relationship between BMI and SES and that low school SES and rural locality of the school are school-level risk factors of child obesity (65). Thus, we recommend family-based education on healthy eating and exercise. In families with insufficient investment in education and poor income, children's obesity reduction is difficult because of the restrictions of SES factors; thus, children from lower-SES families tend to eat excessive and poor-quality foods, including fried foods. Therefore, we confirm that children from lower-SES families are more vulnerable to obesity risk than other children, especially in China's rural and western areas. Thus, related policies targeting to improve the equitable development of SES factors are strongly recommended.

A study finds that majority of the inequality in childhood obesity is explained by parental socioeconomic gradients (67), with socioeconomic factors accounting for 75.8% of the existing inequalities. Residential areas and education provided by the mother were the most important causes of inequality (68). It is important to tailor policies that target child obesity/overweight to tackle not only the prevalence of this disease but also its distribution (69). The results of our study support the findings discussed above. Our study also confirms that the effects of family income and education significantly dominate child obesity inequalities. The results indicate that inequalities in socioeconomic factors measured by family income play a critical role in mitigating the obesity rate among rural and left-behind children, while income differences contribute to the obesity inequalities among left-behind and non-left-behind children. In our analysis, the level of family education is an important contributing factor that increases the obesity inequalities between urban and rural children. In addition, family income and education are the major contributors to changes in the obesity differences between left-behind and non-left-behind children. Thus, we believe that childhood obesity differences are rooted in socioeconomic inequalities, including urban–rural shocks, left-behind shocks, and family capital. Population-based interventions are important in preventing the prevalence of childhood obesity, but policies that target vulnerable groups are also needed to alleviate the inequalities in SES factors.

A study finds that the experience of being left behind or of parents' migration may reduce the care provided to Chinese children and thus affect the health of children (70). Kristin (71) confirms that cumulative adverse childhood experiences may exacerbate existing social disparities in children's health. Jessica et al. (72) confirm that rural children experience health and healthcare disparities compared with their urban peers and represent a unique and vulnerable pediatric patient population. The root causes of health inequalities (obesity, anxiety, infectious diseases, injuries, prematurity, and low birth weight) of children are complex, and interventions to address child health inequalities must consider the structural determinants (73). We also find that the negative experience of being left-behind has a detrimental consequence for child physical health, and grandchild care is significantly associated with child obesity because of the early negative experience of parental absence. Thus, it is necessary to take effective family-level measures to decrease childhood obesity caused by cumulative adverse experiences.

Some scholars claim that children's age and gender are risk factors of childhood obesity (74). We document that gender and age are significantly associated with child obesity and its inequalities among Chinese school-age children and adolescents.

This study has several notable strengths. We apply the new determination criteria using ten-year survey datasets to explore the obesity inequalities in China. The findings could help people reduce the intergenerational spoil shock and increase the improvement effect of medical insurance on child health. In addition, this study provides new evidence for the development of population-based interventions and equitable medical insurance policies to prevent the deterioration of child obesity among Chinese school-age children and adolescents. However, some points are worth improving such as data limitations and the lack of information on physical exercise and sleeping habits, as well as parental overweight. Because of the cross-sectional limitation of the data, although this study confirms that children's family environment and SES factors contribute to child obesity differences, these factors cannot explain all the mechanisms of obesity inequalities. Given the complexity of family-based intergenerational care in China, more in-depth analysis is needed in future.

## Conclusion

We document the influences of grandchild care and medical insurance on child obesity using unique CFPS data (2010–2020). Our results indicate that the epidemiological status of obesity among Chinese school-age children and adolescents has become more severe than in the last decades. Thus, measures that prevent and control the epidemiological trend are urgently needed. We confirm that child obesity among Chinese school-age children and adolescents is caused by many risk factors, including family characteristics, SES factors, and genetic influence. Grandchild care is significantly positively associated with child obesity, while the implementation of medical insurance can decrease the probability of being obese for Chinese school-age children and adolescents. Our study has policy implications. Specifically, improving the medical insurance coverage of children is likely to alleviate the obesity risks among Chinese school-age children and adolescents.

Another significant finding of this study is that inequalities in obesity are rooted in socioeconomic environmental differences and policy efficiency, including household education, income, urban–rural segmentation, and left-behind risk shocks. Using BO to decompose childhood obesity inequalities, we find that the role of grandchild care and public medical insurance and grandchild care may increase the obesity inequalities between urban and rural children, while grandchild care may increase the obesity inequality between non-left-behind and left-behind children because of the shock of parental absence. With the gradual widening of the gap between urban and rural areas in China, the urban–rural structural barriers may set an obvious barrier for the health equality and obesity prevention among different Chinese children. More importantly, children from urban areas can efficiently enjoy better educational resources and sound medical systems than rural children. We recommended tailoring policies to target child obesity and tackle not only the prevalence of obesity but also its distribution. Therefore, it is important to break down the barriers between urban and rural areas and ensure the fairness in public healthcare resource provision and medical accessibility. We should ensure that children from the rural areas and left-behind families equally benefit through a renewed focus on inequalities.

We suggest that relevant government departments comprehensively consider the effects of these risk factors and develop a systematic family risk assessment tool for child obesity among Chinese school-age children and adolescents. First, family-level public intervention measures should be implemented among Chinese school-age children and adolescents. The vulnerable children should be made a priority for obesity prevention, especially among rural and left-behind children. Governments at different levels should optimize the school-based obesity prevention program and strengthen the comprehensive family support mechanism to develop a health-supported environment for child obesity prevention. On the one hand, the government should improve the unified standards of diagnosis, assessment, treatment, prevention, and management of child obesity for primary care providers. On the other hand, health education, diet control, and scientifically planned exercise regimens should be promoted among Chinese school-age children and adolescents. Moreover, the quality and efficiency of children's healthcare must be improved to prevent the factors caused by school lockdown and home isolation during the COVID-19 outbreak. Because of the potential absence of parental supervision and the affordability of healthcare resources, it is advised to improve the quality of grandchild care among Chinese school-age children and adolescents. For obese children, a comprehensive and systematic intervention approach is needed, focusing on a multi-level intervention model and fully strengthening the responsibilities of families, schools, medical institutions, and the government, to achieve the goal of preventing and controlling child obesity. The government can also help prevent obesity by purchasing services and allowing relevant social organizations to provide professional care services for rural and left-behind children.

Second, we recommend that the multi-level medical insurance system be designed to alleviate the obesity illness's economic burden by constructing a more inclusive medical insurance system for the affected children. In addition, clinicians should conduct an overall assessment of the obesity risk factors and then prescribe a holistic approach for obesity treatment of these children. Moreover, considering the difficult situations of insured children, the government can explore the integrated medical insurance systems, establish a gradient commercial medical insurance plan for different groups of children, and focus on improving the coverage scope of the medical insurance system.

Third, the obesity inequalities should be alleviated by improving the SES of disadvantaged families and gradually integrating the effects of the medical insurance systems. Some related social policies targeted at child obesity inequalities among Chinese school-age children and adolescents are advised as follows: (1) increasing the financial input in child healthcare and implementing a mutual fund system specifically for these vulnerable groups; (2) improving nutrition, the health environment, and medical conditions for disadvantaged children to avoid obesity inequalities due to urban–rural and left-behind experiences; (3)improving the fairness of basic medical insurance and scientifically defining the reimbursement scope of children's medical insurance systems; (4) establishing a laddered policy rate of reimbursement to address SES inequalities and providing care subsidy for grandchild-care families in need; (5) improving the left-behind children's medical accessibility of healthcare insurance payments in different-grade hospitals and popularizing children's commercial medical insurance system; and (6) promoting the social environment to prevent and control the prevalence of child obesity.

## Data availability statement

The original contributions presented in the study are included in the article/supplementary material, further inquiries can be directed to the corresponding authors.

## Ethics statement

The studies involving human participants were reviewed and approved by Peking University Biomedical Ethics Committee (No. IRB00001052-14010). Written informed consent to participate in this study was provided by the participants' legal guardian/next of kin. The animal study was reviewed and approved by Peking University Biomedical Ethics Committee (No. IRB00001052-14010). Written informed consent was obtained from the individual(s), and minor(s)' legal guardian/next of kin, for the publication of any potentially identifiable images or data included in this article.

## Author contributions

JY: conceptualization, methodology, validation, formal analysis, reviewing, and editing. YS: conceptualization, methodology, validation, writing, reviewing, and editing. YD: conceptualization, methodology, validation, formal analysis, and funding acquisition. ZL: conceptualization, reviewing and editing, supervision, and funding acquisition. All authors contributed to the article and approved the submitted version.

## Funding

This research was funded by the National Social Science Foundation of China (Grant No. 21CSH011) and Qian Duan-sheng Outstanding Scholars Support Program of China University of Political Science and Law. This work was supported by CFPS of the Institute of Social Science Survey of Peking University. The data from China Family Panel Studies (CFPS), is funded by Peking University.

## Conflict of interest

The authors declare that the research was conducted in the absence of any commercial or financial relationships that could be construed as a potential conflict of interest.

## Publisher's note

All claims expressed in this article are solely those of the authors and do not necessarily represent those of their affiliated organizations, or those of the publisher, the editors and the reviewers. Any product that may be evaluated in this article, or claim that may be made by its manufacturer, is not guaranteed or endorsed by the publisher.

## Footnotes

^1^See https://stats.oarc.ucla.edu/r/dae/mixed-effects-logistic-regression/.
